# Assessment of the Physicochemical Properties of Ultrafine Particles (UFP) from Vehicular Emissions in a Commercial Parking Garage: Potential Health Implications

**DOI:** 10.3390/toxics12110833

**Published:** 2024-11-20

**Authors:** Nachiket Vaze, Leonardo Calderon, Irini Tsiodra, Nikolaos Mihalopoulos, Charles N. Serhan, Bruce D. Levy, Philip Demokritou

**Affiliations:** 1 Nanoscience and Advanced Materials Center, Environmental and Occupational Health Sciences Institute, Rutgers University, Piscataway, NJ 08854, USA; nv282@rutgers.edu (N.V.); ldc71@eohsi.rutgers.edu (L.C.); 2 Institute for Environmental Research and Sustainable Development, National Observatory of Athens, Lofos Koufou, P. Penteli, 15236 Athens, Greece; itsiodra@noa.gr (I.T.); nmihalo@noa.gr (N.M.); 3 Environmental Chemical Processes Laboratory, Department of Chemistry, University of Crete, 71003 Heraklion, Greece; 4 Center for Experimental Therapeutics and Reperfusion Injury, Department of Anesthesiology, Perioperative and Pain Medicine, Hale Building for Transformative Medicine, Brigham and Women’s Hospital and Harvard Medical School, Boston, MA 02115, USA; cserhan@bwh.harvard.edu; 5 Pulmonary and Critical Care Medicine, Department of Medicine, Brigham and Women’s Hospital and Harvard Medical School, Boston, MA 02115, USA; blevy@bwh.harvard.edu

**Keywords:** ultrafine particles, vehicular emissions, garage pollution, particle lung deposition

## Abstract

Vehicular emissions are a major culprit in the rise of urban air pollution. The particulate matter (PM) emitted from vehicular sources includes primarily ultrafine particles (UFPs) with aerodynamic diameters less than 0.1 µm (PM_0.1_) and is linked to adverse respiratory and cardiovascular health effects. Despite this knowledge, few exposure assessment studies exist that detail the physicochemical properties of PM in parking garages. In this study, airborne PM emitted by vehicles in a parking garage of a hospital in New Jersey was sampled, during winter and summer seasons, and physicochemically characterized. The results indicate that the mass concentrations of the UFPs in the garage were 2.51 µg/m^3^ and 3.59 µg/m^3^, respectively. These UFPs contained a large percentage of elemental carbon and toxic elements. They also contained polycyclic aromatic hydrocarbons (PAHs), having deleterious health effects. An inhalation particle modeling revealed that 23.61% of these UFPs are deposited in the pulmonary region of the lung, translating to a dose of 10.67 µg for winter and 15.25 µg for summer, over a typical 40 h work week. These high deposited levels of UFPs and their complex chemistry levels further warrant the need for toxicological assessment of UFPs related to vehicular emissions.

## 1. Introduction

Particulate matter (PM) is a principal component of both indoor and outdoor air pollution and has been associated with negative health outcomes [1,2]. It has also been implicated as a major driver of climate change, making it a global concern [3,4,5]. Ambient PM consists of a mixture of solid and liquid particles of various sizes suspended in air, emitted from various sources with complex chemistry [6]. There are various sources of PM such as natural sources, industrial processes, fuel burning, wildfires, and vehicular emissions [3]. PM consists of particles that range from larger particles with diameters up to 100 µm to particles with an aerodynamic diameter of 0.1 µm or less (PM_0.1_) which are also termed as ultrafine particles (UFP). The chemistry of PM is source dependent and contains both inorganic and organic compounds [3].

Of the various PM sizes and size fractions, PM_2.5_ (particles with diameters less than 2.5 µm) is a size fraction of PM that has been extensively studied in a plethora of epidemiological and toxicological studies for its adverse effects [7,8]. PM_2.5_ can penetrate into the lungs and enter the bloodstream causing respiratory, cardiovascular, and other adverse health effects such as asthma, bronchitis, and lung cancer [9,10,11]. Thus, the PM_2.5_ has been monitored and regulated widely across the globe with regulations from the World Health Organization (WHO) as well as the US Environmental Protection Agency (EPA) [12]. Recently, the EPA lowered the National Ambient Air Quality Standards (NAAQS) for PM_2.5_ from 12 to 9 µg/m^3^ to protect vulnerable populations from respiratory and other diseases [13].

Despite the focus on public health measures to monitor and control PM_2.5_, there has not been similar attention paid to the smaller PM_0.1_ (UFP) particles [14]. UFPs’ smaller size and larger surface area per mass, compared to the larger inhalable particles, leads to them penetrating deep into the lungs more easily, reaching all the way into the pulmonary alveoli [15,16]. Furthermore, their large surface area per volume and complex chemistry lead to an increased level of toxicity [17].

Historic and emerging epidemiological and toxicological data indicate that exposure to ambient UFPs is linked to respiratory diseases such as asthma, chronic obstructive pulmonary disease (COPD), and cardiovascular diseases, owing to UFPs ability to instigate pulmonary accumulation, lung injury, and inflammation and systemic translocation within the body system [18,19,20]. Traffic-related sources are a significant contributor of UFP pollution, particularly in urban environments and major cities [21,22,23,24]. UFP can be either from exhaust-related causes, such as complete and incomplete combustion, or from non-exhaust-related sources, such as wildfires, volcanic eruptions, and mechanical abrasion of brakes and tires. A study conducted by the authors during the Canadian wildfires of 2023 found that UFPs generated in the atmosphere during peak phase were at a high concentration of 145.8 μg/m^3^. In terms of UFPs generated by vehicular processes, Dahl et.al., speculate that studded tires, used in cold climates to improve grip, generate ultrafine particles. Singh et.al. documented~5 ×10^6^ particles/cc levels of UFP generation from toner cartridges [4,25,26,27,28].

Epidemiological data suggest that traffic-related air pollution is a risk factor for wheezing, asthma prevalence, and allergic sensitization [29,30], with birth cohorts showing positive correlations between exposure to traffic pollution and atopic diseases and allergic sensitization in children (reviewed in [31,32]). With increased urbanization, human exposures to traffic-emitted UFPs are increasing. Exposure to UFPs increases the risk of asthma exacerbation and severity, including to the common household allergen house dust mite (HDM) [33]. Recently, the American Thoracic Society and the American Academy of Allergy, Asthma, and Immunology emphasized the importance of research on potential adverse health effects of UFP exposure [34,35]. An important factor to consider for evaluating the negative health impacts is the dependence of exposure to seasonal variations in the UFP generated [36,37].

Previous research has primarily focused on vehicular emissions from roads or open areas, where particles from other sources such as wildfires may alter the physicochemical properties of PM_0.1_ originating from vehicle emissions [38,39,40]. Although there are a few studies on the assessment of PM in garages, which are dominated by vehicular emissions [41,42,43], data on the physicochemical assessment of such PM are very rare and are desperately needed by public health assessors given that there are people working in garages and their exposure may cause adverse health effects.

In this study, the parking garage of a hospital was chosen to collect size-fractionated PM during winter and summer seasons, and the collected PM was assessed for its physicochemical properties using state-of-the-art analytical methods and real-time aerosol instrumentation. PM inhalation modeling was also used to estimate the delivered to the respiratory tract UFP dose.

## 2. Materials and Methods

**Figure 1:**
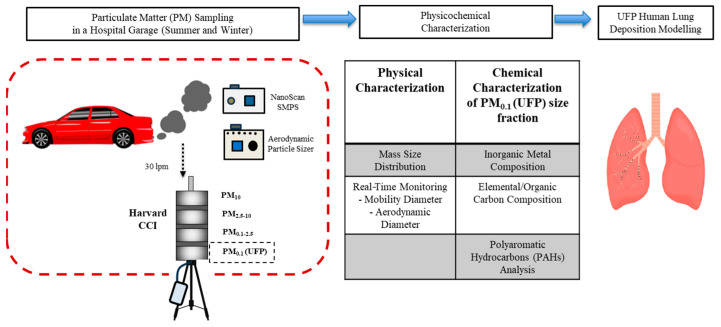
Overall schematic of this study. The three major steps were the collection of size-fractionated PM from a hospital garage, physicochemical characterization of the collected PM, and the assessment of the deposition in human lungs of the UFP fraction.

Figure 1 summarizes the study design. In summary, PM was sampled over two distinct sampling periods, conducted in the winter of 2023 and in the subsequent summer of 2024 in a hospital garage. Each of the two sampling periods was broken down into a series of six (6) multi-day individual campaigns. The sampling was conducted using time-integrated PM sampling systems developed by the authors [44]. Size-fractionated PM was used for offline physicochemical characterization using state-of-the-art analytical methods such as Inductively Coupled Plasma Mass Spectrometry (ICP-MS) and Gas Chromatography Mass Spectrometry (GC-MS) to assess its chemical composition. The mass size distribution was calculated for the size fractions of PM_0.1_, PM_0.1–2.5_, PM_2.5–10_. Real-time PM monitoring systems were also used to measure the particle number concentration as a function of size.

A particle lung deposition model was then utilized to assess the UFP dose deposited in human lungs, as a function of exposure time. The size-fractionated PM was also extracted from the filters using methods developed by the authors [45,46] and stored in a PM repository for use in future toxicological studies. The details of the methodology are as follows:

### 2.1. Garage Sampling Site

The selected location is quaternary care hospital in central New Jersey. The garage is a three-level semi-enclosed facility with the lowest (third) level below ground floor being fully enclosed underground. The garage is mechanically ventilated. A PM sampling site was chosen on this lower level, a location that experienced the highest traffic flow, as it was centrally located on the vehicles’ path out of the garage. This garage accommodates approximately 1000 cars entering and exiting daily. The PM sampling equipment was installed at a sampling height of 1.5 m above ground level and operated continuously. The winter period sampling was conducted from 5 February 2024 to 12 April 2024. The summer period sampling was conducted from 29 May 2024 to 5 August 2024.

### 2.2. PM Size-Fractionated Sampling Using Time Integrated Equipment

The Harvard Compact Cascade Impactor (CCI) developed by the authors was used for size-fractionated sampling of inhalable PM in various aerodynamic size fractions, namely PM_0.1_, PM_0.1–2.5_, PM_2.5–10_ [44]. UFPs (PM_0.1_) were collected on inert, chemically washed Polytetrafluoroethylene (PTFE) membrane disc filters (PTFE membrane disc filter: 47 mm diameter, 2 μm pore size, Pall Corporation, Port Washington, NY, USA). Other PM size fractions were collected on chemically cleaned polyurethane foam (PUF) substrates as in recent publications by the authors [46]. Teflon filters and PUFs were pre- and post-sampling acclimated for 48 h in controlled temperature and relative humidity (RH) conditions (20–23 °C and 30–40% RH) and were weighed to derive PM mass size concentrations across size fractions. Teflon filters containing the PM_0.1_ were used for ICP-MS analysis.

The size-fractionated PM collection ‘campaigns’ were performed in a multi-day fashion with the flow of CCI monitored and adjusted every 24 h to its nominal 30 L per minute (lpm) flow rate. These flow rate measurements were conducted in triplicate. If the flow rate was found to be below 95% of the 30 lpm operating flow rate of the HCCI (corresponding to 28.5 lpm), the sampling campaign was concluded and filters/PUFS were removed for further processing. This time period was considered one ‘campaign’. Next campaign was initiated and same protocol was followed. Multiple CCIs were used in parallel for each sampling campaign, all co-located. For each size fraction, 16 samples were collected for winter, and 18 for summer.

### 2.3. Real-Time Monitoring of PM

To determine the particle number concentration of PM as a function of size, the Nanoscan Scanning Mobility Particle Sizer (SMPS, model 3910, TSI Inc., Shoreview, MN, USA) and the Aerodynamic Particle Sizer (APS) Model 3321 (TSI Inc., Shoreview, MN, USA) were utilized. The Nanoscan instrument measures particle sizes from 10 to 420 nm and the APS instrument covers a size range of 0.5 to 20 µm in aerodynamic diameter. The real-time monitoring was performed on a single day during the overall sampling period to derive the PM size distribution and particle number concentration as a function of size.

### 2.4. Inorganic Elemental Analysis Using Inductively Coupled Plasma Mass Spectrometry (ICP-MS)

Samples were digested using a mixed-acid microwave-assisted Teflon-bomb digestion method (Milestone Ethos Easy SK15 with enclosed microvials). Liquid samples were pipetted into the vials and weighed, while filter membrane samples were placed in the vials with the support ring removed. Each vial received the following acid mixture: 1.50 mL of 16 M Nitric Acid, 0.5 mL of 12 M Hydrochloric Acid, and 0.2 mL of 28 M Hydrofluoric Acid. The bombs were then sealed with a surrounding acid of 16 M Nitric Acid to pressurize the vessels. The contents were heated to 195 °C, with a 20 min ramp-up to 195 °C, followed by a 15 min hold at that temperature. Then, the samples were diluted to a target volume of 17 mL using ASTM (American Society for Testing and Materials) Type 1 water, and the final volumes were calculated gravimetrically. Digested samples were diluted for analysis using SF-ICP-MS. The analyses were conducted with a magnetic-sector ICP-MS (Thermo Element XR), scanning a variety of isotopes at different resolutions. Sample concentrations were corrected for blanks using the mean method blank of the batch. More details can be found in the authors’ previous publications [27,28].

### 2.5. Assessment of Elemental and Organic Carbon Composition

Elemental carbon (EC) and organic carbon (OC) are fundamental components of particulate matter (PM) in the atmosphere, originating from both natural and anthropogenic sources. EC/OC analysis is a method used to quantify these components within atmospheric aerosols, leading to a better understanding of air quality and pollution sources [47]. Pre-baked quartz filters (Pallflex Tissuquartz filter: 47 mm diameter, Pall Corporation, Port Washington, NY, USA) were used for the analysis of EC/OC ratio of PM_0.1_ size fraction. The protocol employed for this was the NIOSH 5040 method [48]. The filters were physically cleaned before the collection following the NIOSH 5040 protocol (800 Celsius for 5 h). The filters were pre- and post-sampling acclimated for 48 h in controlled temperature and relative humidity (RH) conditions (20–23 °C and 30–40% RH) and were weighed in triplicates. The samples were acquired thus, followed by a thermal-optical procedure to distinguish between EC and OC. The thermal-optical transmittance (TOT) technique, a widely used method, was employed. This technique heats the sample in an inert atmosphere to volatilize OC, followed by an oxidizing atmosphere to combust EC. Throughout this process, a laser beam monitors the filter transmittance to accurately differentiate between the OC and EC fractions. The limit of detection for the results was 38 ng/m^3^. More details can be found in the authors’ previous publications [28,46,49].

### 2.6. Polyaromatic Hydrocarbons (PAHs) Analysis

For PAH analysis, the protocol described in Tsiodra et al. [50] was used with slight modifications. Pre-baked quartz filters (Pallflex Tissuquartz filter: 47 mm diameter, Pall Corporation, Port Washington, NY, USA) were used for the PAH analysis of PM_0.1_ size fraction. The protocol employed for this was the National Institute for Occupational Safety and Health (NIOSH) 5040 method [48]. The filters were physically cleaned before the collection following the NIOSH 5040 protocol (800 Celsius for 5 h). The filters were pre- and post-sampling acclimated for 48 h in controlled temperature and relative humidity (RH) conditions (20–23 °C and 30–40% RH) and were weighed in triplicates. Samples were spiked with a known quantity of deuterated PAHs (16 members) mixture (CPAchem Ltd., Bogomilovo, Bulgaria). These members were used as surrogate standards for the identification of PAHs for the calculation of the recovery efficiencies and were added prior to extraction. PAHs were extracted using pressurized liquid extraction with an accelerated solvent extractor system (Dionex ASE 300; Thermo Fisher Scientific Inc., Waltham, MA, USA)) and solvent mixture 50:50 *n*-hexane-dichloromethane. The sample extracts were purified through a silica column and the selected fractions were collected using different polarity solvents. The fraction which contained PAHs was eluted using 11 mL of *n*-hexane/ethyl acetate (8:2 *v*/*v)* and was condensed to the reduced volume of 0.1 mL. At the end of the experiment, specific amount of [^2^H_12_]perylene was added as internal standard. During the day of the analysis, to quantify the desirable compounds, a mixture of native and deuterated PAHs was injected to the gas chromatography/mass spectrometry (GC/MS) instrument for the calculation of the relative response factors (RRF). Analysis was performed using an Agilent 7890 GC (Agilent Technologies Inc., Santa Clara, CA, USA) equipped with an HP-5MS capillary column (30 m × 0.25 mm i.d. × 0.25 μm phase film) and an Agilent 5975C mass selective detector.

The calculations of carcinogenic risk related to PAH inhalation exposure (BaPeq) were performed based the toxic equivalent factor (TEF) approach (Taghvaee et al., 2018) [51]. The TEFs represent the toxicity of each congener relative to the reference toxicity of Benzo (a)pyrene which is the only IARC (International Agency for Research on Cancer) group I carcinogenic PAH. The calculated value is made based on Equation (1):

(1) $$\sum {BaP}_{eq}=\sum (C_{i}\;{TEF}_{i})$$

where C*_i_* is PAH concentration (ng m^−3^) and TEF*_i_* is the Toxicity Equivalent Factor of each PAH member (i).

### 2.7. UFP Lung Deposition Modeling

The Multiple-Path Particle Dosimetry (MPPD) model is an important numerical tool utilized for predicting the deposition of particles inside the human respiratory tract [52]. This model simulates the behavior of an inhaled particle based on parameters such as particle size, shape, and density, and human breathing patterns. It provides deposition across three major regions of the human respiratory tract [53,54]. In this study, the MPPD V3.04 software (ARA, Albuquerque, NM) was utilized. The UFP aerosol concentration obtained from the mass size distribution results for both the winter and summer campaigns was used as input aerosol concentration. Earlier studies from the authors have demonstrated that for inhaled nanoparticles, instead of using bulk density (ρ_b_), the use of the more realistic effective density (ρ_eff_) is essential to determine a more accurate lung deposition and dosimetry [55]. Hence, to determine the ρ_eff_, a study by Rissler et al., where they calculated the ρ_eff_ of diesel vehicle-emitted black carbon to be 0.6, was utilized [56]. It is worth noting that the effective density is not the actual density of the PM and differs significantly due to its fractal-like structure as shown in a study recently published by the authors [55]. Finally, the MPPD calculations for human exposure were performed using the Yeh/Schum symmetric model with a functional residual capacity of 3300 mL and head volume of 50 mL [57]. The nasal respiratory rate (RR) was set to 12 breaths/minute, the tidal volume (TV) to 625 mL, and the inspiratory fraction to 0.5 [57].

### 2.8. Statistical Analysis

All filter weights and flow rates were measured in triplicate. Flow rates measured and time sampled for the duration of the campaign were utilized to calculate total volume, for the determination of the mass size concentration. Averages and standard deviations were calculated across PM mass concentrations obtained by each HCCI. The student’s *t*-test was utilized for obtaining *p*-values. For NanoScan measurements, averages of 100 continuous measurement were plotted, along with ±1 standard deviation as the error. For the APS measurement, averages of 200 such measurement were plotted, along with ±1 standard deviation as the error. All instruments were calibrated before use.

## 3. Results and Discussion

### 3.1. Size-Fractionated PM Mass Concentrations

**Figure 2:**
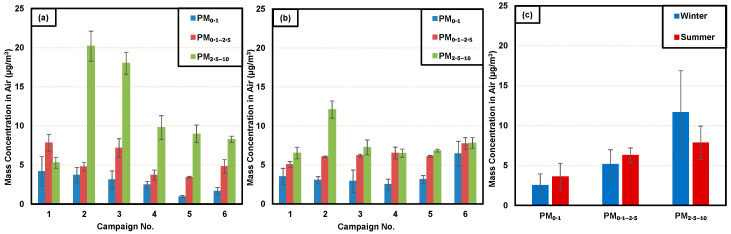
Time-integrated PM mass concentration as a function of aerodynamic size, as denoted for (**a**) each winter sampling campaign; (**b**) each summer sampling campaign; (**c**) comparison of average concentrations over the entire winter and summer sampling periods. The error bars denote ±1 standard deviation between concentrations obtained from individual HCCIs (*n* is the number of CCIs involved for each sampling period with *n* = 16 for winter, *n* = 18 for summer).

A campaign-wise breakdown of the mass size PM concentration for the various size fractions is shown in Figure 2a (for the winter sampling period) and Figure 2b (for the summer sampling period).

For sampling during the winter period, the following results were obtained (Figure 2a). For the PM_0.1_ size (UFP), the maximum concentration obtained was 4.17 (±1.87) µg/m^3^ during the first campaign. Similarly, for the PM_0.1–2.5_, the maximum concentration obtained was during the first campaign at 7.82 (±1.08) µg/m^3^. This was the highest concentration obtained for this size fraction across all of the winter and summer sampling campaigns. For the PM_2.5–10_ size fraction, campaign #2 produced the highest levels observed with 20.18 (±1.92) µg/m^3^. This is the highest mass concentration obtained for this size fraction across any of the summer or winter campaigns.

For summer period sampling (Figure 2b), the maximum PM_0.1_ size (UFP) concentration obtained was 6.44 (±1.58) µg/m^3^, during the sixth and final campaign. This is the highest concentration of UFP sampled during any campaign for the winter or summer periods. For the PM_0.1–2.5_, the maximum concentration obtained was also during the sixth campaign, with 7.73 (±0.76) µg/m^3^. For the PM_2.5–10_ size fraction, the highest concentration was obtained during the second campaign, 12.09 (±1.11) µg/m^3^.

Figure 2c summarizes the average mass concentration of PM for each inhalable size fraction for the winter and summer periods (all six campaigns averaged). The seasonal variability of the PM generated can be observed here for all size fractions considered. The average concentration of PM_0.1_ size (UFP) was 2.51 (±1.43) µg/m^3^ during the winter and increased to 3.59 (±1.67) µg/m^3^ during the summer (*p* = 0.0527). The concentration of PM_0.1–2.5_ was 5.14 (±1.82) µg/m^3^ during the winter and 6.27 (±0.92) µg/m^3^ during the summer (*p* = 0.027). For the PM_2.5–10_**,** it was 11.62 (±5.24) µg/m^3^ during the winter and 7.83 (±2.1) µg/m^3^ during the summer (*p* = 0.008). For the two smaller size fractions of PM, the concentrations were higher during the summer, whereas for the larger PM_2.5–10_ size, winter concentrations were higher, indicative of a seasonal variability.

**Figure 3:**
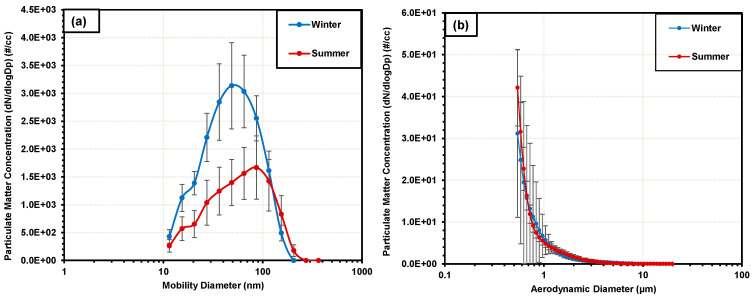
Real-time monitoring of PM particle number concentration as a function of size emissions in the garage conducted during winter and summer campaigns: (**a**) mobility size distribution utilizing a TSI Nanoscan SMPS and (**b**) aerodynamic size distribution using TSI APS. Error bars denote ±1 standard deviation.

It is worth noting that in terms of the PM mass concentrations, the US EPA guidelines for PM_2.5_ and PM_10_ exposure for a 24 h average are 35 and 150 µg/m^3^, respectively [13]. The levels observed in this study are lower than these exposure standards; however, no regulations exist as to the exposure limits for UFP. In addition, compared to the larger particles of the PM_2.5_ size (particles in the PM_0.1–2.5_ size range), UFPs have miniscule mass but greater particle number concentration, as shown in the real-time data in this study (see Figure 3). Being nano-sized particles, they also have significantly higher surface area.

Comparing these results to literature, in a study conducted in completely closed parking garages in Belgium, the concentration of PM_2.5_ and PM_10_ was found to be 43 (±3) µg/m^3^ and 58 (±13) µg/m^3^, respectively [42]. In present study, the authors did not detect such high concentrations, which could be attributed to the semi-enclosed nature of the parking garage and mechanical ventilation. In another study involving an underground hospital parking garage in Spain, the PM_2.5_ measured was 6.29 µg/m^3^, which is a similar value to the PM_0.1–2.5_ concentration observed by the authors during summer season, and slightly higher than those observed during winter [41].

There have only been a few studies that assessed the seasonal variability of traffic-related UFP. A study of roadside sampling from Delhi, India noted that the UFP concentrations during the winter period ranged from 2.8 to 10.3 μg/m^3^ during the evening traffic periods and during summertime the concentrations had lesser variability, ranging from 2 to 5 μg/m^3^. By contrast, the authors in the present study found the average winter concentrations of UFP to be lower than those during the summer [58].

### 3.2. Real-Time Measurement of Particle Number Concentration as a Function of Size

Real-time PM monitoring was conducted for two hours each on a chosen date, during the winter and summer periods, to assess the particle number concentration as a function of particle size. These results were considered a snapshot of the expected particle concentrations in the garage.

The measurement of UFP particles utilizing the Nanoscan SMPS for both the winter and summer sampling periods is illustrated in Figure 3a. For winter, the geometric (GMD) and count mean (CMD) mobility diameters of the particulate matter were 46.5 (±1.85) nm and 55.84 (±2.26) nm, respectively, indicative of the nanoscale nature of emitted PM. The average concentration of particles across the entire size range was 1.88 (±0.3) ×10^4^ particles/cc. The highest concentration of particles was observed at the 48.7 nm size with 3137.87 (±774.57) particles/cc. All particles sampled were within the range of 11 nm–154 nm and no particles were detected at the largest sampling sizes of the Nanoscan SMPS instrument at 205.4, 273.8, and 365.2 nm.

For sampling during the summer, the geometric (GMD) and count mean (CMD) mobility diameters of the particulate matter were 55.16 (±4.19) nm and 60.04 (±6.22) nm, respectively. The total concentration of particles was 1.08 (±0.35) × 10^4^ particles/cc. The highest concentration of particles was observed at the 86.6 nm size with 1667.54 (±567.84) particles/cc.

A recent study of traffic-related PM measured roadside reported a similar trend of sizes, with an average GMD of 49.2 nm during the winter and 59.8 nm during the summer [59]. Ragettli et.al. assessed commuter exposure to UFP in different urban locations and found the particulate matter GMD for the winter and summer to be 49 and 58 nm, respectively, similar to the results presented here [60].

Figure 3b summarizes the larger size particles (0.542–20 µm), as measured with the Aerodynamic Particle Sizer (APS). Here, for the winter, the highest concentration obtained was for the lowest size measured, 542 nm, with 31.14 (±20) particles/cc, with the concentration decreasing continuously as higher sizes were sampled. For summer sampling, a similar trend was observed, with the highest concentration at 542 nm with 42.12 (±9.13) particles sampled. This is expected and it is known that vehicular PM emissions are dominated by nanosized particles with only a few larger particles primarily emitted from other ambient sources [22].

As stated earlier, there are only a few studies that focus on vehicular PM emissions in public garages. For example, a study that measured exposure for mechanics in a garage to PM_1_ found a particle number concentration of 7.6 (±0.02) × 10^4^ particles/cc [61]. In another study, Obaidullah et al. measured overall particle concentration and size distribution of PM in a garage ranging from 6 nm to 10 µm and measured 2.8–4.7 × 10^4^ particles/cc [42]. A hospital garage-based study from Spain reported overall levels of PM_10_ at 8 × 10^4^ particles/cc [41]. These values are similar to the concentrations obtained by the authors here.

It is worth noting that observed seasonal variations in the physicochemical properties of UFPs could be because even in mechanically ventilated garages like this one, large amounts of ambient air are circulated for ventilation purposes; therefore, any seasonal variability from other ambient UFP sources such as wildfires will affect the UFPs sampled in the garage. It is also known that particle emissions from combustion engines can also be affected by ambient air temperatures [62,63].

### 3.3. Inorganic Metal Analysis of UFP

**Figure 4:**
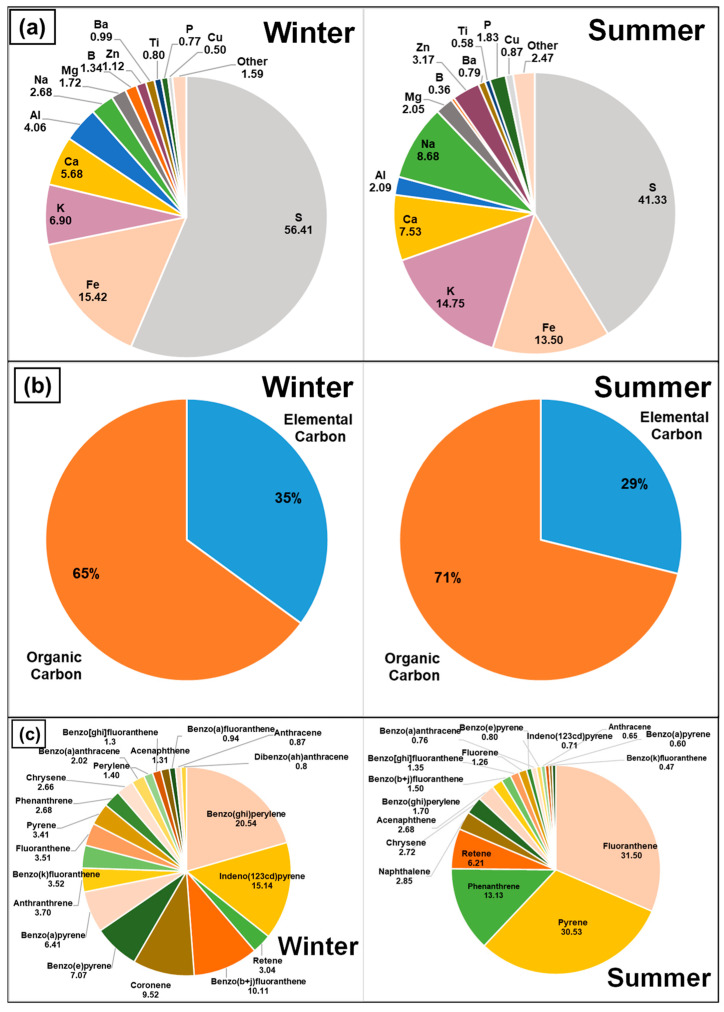
Chemical characterization of the UFP size fraction of the PM sampled from the garage. The results are represented as percentages for (**a**) Inorganic Metal Analysis, (**b**) Elemental and Organic Carbon Composition Analysis and (**c**) Polycyclic Aromatic Hydrocarbons (PAHs) Analysis. The results are represented as weight over weight fraction percentages (*w*/*w*)%.

Inductively Coupled Plasma Mass Spectrometry (ICP-MS) was utilized to detect inorganic elements for the PM_0.1_ size fraction of the collected particles. The results of the weight over weight (*w*/*w*) percentage composition of various elements are shown in Figure 4a as weight over weight for the elements analyzed.

For the UFP collected during the winter, the largest element found was Sulfur at 56.41%, followed by 15.41% Iron and 6.9% Potassium. Calcium was 5.68%, followed by Aluminum at 4.05% and 2.68% Sodium. Magnesium, Boron, and Zinc were detected at levels of 1.72, 1.34, and 1.12%, respectively. Other elements such as Barium, Titanium, Phosphorous, and Copper were detected at lower than 1% levels.

For the UFPs collected during the summer, Sulfur was the biggest component at 41.33%. The next highest percentages observed were for Iron at 13.5% and Potassium at 14.75%. Other elements detected were Calcium, Aluminum, Sodium, and Magnesium at 7.53%, 2.09%, 8.68%, and 2.05%, respectively. Zinc was detected at 3.16%, and Barium, Titanium, Phosphorous, and Copper were detected at lower than 1% levels.

Sulfur being the highest elemental component is to be expected as it is emitted primarily from emissions of fuel, motor oil, as well as additives such as zinc dithiophosphate [64]. The higher Sulfur levels during winter sampling observed here have also been observed by Kuwayama et al. in their study of UFPs measured over a yearly cycle [65]. The Potassium percentage fraction was significantly larger in the summer as opposed to in the winter. Zinc was detected twice as much during the summer as opposed to the winter. Zinc is often associated with tire wear and since higher rates of tire wear occur in summer, the higher percentage of Zinc is expected [66]. A review by Moreno-Rios et al. discusses PM_0.1_ from various emission sources and elemental compositions of such. The studies detailed in their review indicate that UFP from vehicular emissions were found to contain elements such as Ag, Al, As, Ba, Be, Ca, Cd, Co, Cr, Cu, Fe, K, Mg, Mn, Mo, Na, Ni, Pb, Pd, Pt, Rh, Rb, Sb, Se, Sr, Ti, U, V, and Zn. Interestingly, they did not comment on the Sulfur component of the assessed PM [67].

### 3.4. Elemental and Organic Carbon Analysis

An analysis of carbon mass fractions was carried out for the PM_0.1_ size fraction of the collected particles and the results are presented in Figure 4b. For the winter sampling period, the concentration of the total collected carbon (TC) was 1.5 µg/m^3^. The organic carbon (OC) concentration was found to be 1 µg/m^3^. The elemental carbon (EC) concentration was 0.54 µg/m^3^. In terms of ratios of mass collected, the OC/TC fraction was 65% and the EC/TC fraction was 35%. The EC/OC ratio was 0.53.

During the summer, the concentration of the total collected carbon (TC) was 2.39 µg/m^3^. The organic carbon (OC) concentration was 1.7 µg/m^3^. The elemental carbon (EC) concentration was 0.69 µg/m^3^. In terms of ratios of mass collected, the OC/TC fraction was 71% and the EC/TC fraction was 29%. The EC/OC ratio was 0.4.

The EC/OC ratio observed here was higher for the winter sampling period, as compared to summer sampling. This trend is similar to the study by Kim et al., who studied the seasonal variations in EC/OC from roadside UFPs and report a significantly higher EC/OC ratio for the winter (0.168) to the summer (0.05) [68]. It is noteworthy that the EC/OC levels found in this study are higher, for the winter and summer, than those found in studies which focus on PM from roadside conditions. For example, Kim et al. studied carbonaceous components in UFP from vehicular sources in a roadway environment. Their findings revealed that the EC/OC ratio for UFP ranged from 0.147 to 0.19 [69]. Another study from Vietnam concerning UFP from the roadside measured the EC/OC ratio as 0.26 [70].

Overall, the differences between EC/OC levels measured in road conditions vs. levels measured in this garage study can be attributed to other UFP sources such as wildfire-emitted particles which can be present in ambient traffic environments which is not the case in a garage environment where the emissions are primarily vehicular ones [71]. For example, a study of wildfire-related PM characterization conducted by the authors observed a ten-fold lower EC/OC ratio of 0.043, as compared to the present study [4].

### 3.5. Polycyclic Aromatic Hydrocarbons (PAHs) Analysis

The PAH analysis for the winter and summer sampling periods is shown in Figure 4c. For winter, the largest component identified was Benzo (*ghi*)perylene at 20.54%. It is used as a marker of gasoline-powered vehicle activity, as it has the highest particle-phase emission factor of the 16 priority PAH in light-duty vehicle exhaust [72,73]. The second most common PAH detected was Indeno (1,2,3-cd)pyrene (IP), consisting of 15.14% of the total PAHs. IP is a prominent air pollutant, with sources identified mainly from traffic [74]. In addition, increased relative abundance presents two high molecular weight congeners, Benzo (b + j)fluoranthene and Coronene.

For the summer period, the concentration of PAHs was measured six times higher compared to the winter levels. Regarding the compositional profile of PAHs, high loadings of medium molecular weight members such as Fluoranthene (23%) and Pyrene (22%) were recorded. These species are present in high loadings in traffic-related factors in source apportionment studies [72,75,76].

The diagnostic ratio of PAHs is commonly used in the literature as a source indicator [77]. The diagnostic ratios of Fluoranthene to Pyrene and Indeno (1,2,3-cd)pyrene to Benzo (ghi)perylene were found equal to 0.5 and 0.3–0.4, respectively (for both winter and summer samples), confirming the traffic origin. Furthermore, laboratory studies which characterize the PAH profile from vehicular emissions demonstrate the presence in high loadings of low molecular weight PAHs (with two and three aromatic rings), like Naphthalene, Phenanthrene, and medium molecular weight PAHs such as Fluoranthene and Pyrene in diesel-powered vehicles [78,79]. In addition, emissions from gasoline powered vehicles are enriched with medium and high molecular weight PAHs (Fluoranthene, Benzo (a)anthracene, Indeno (1,2,3-cd)pyrene, Benzo (ghi)perylene and Coronene [80,81]. In the present study, the same species were also detected in high frequencies in both seasons.

### 3.6. Respiratory PM_0.1_ Deposition Modeling

**Figure 5:**
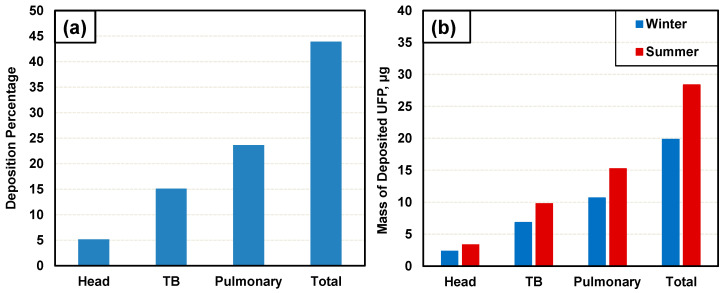
Deposition of UFPs in the head, tracheobronchial (TB), pulmonary, and total region of the human respiratory tract derived by MPPD model. Results represented in terms of (**a**) the percentage of UFP aerosol deposited in each region of human respiratory tract and (**b**) mass of UFPs deposited in each region of human respiratory tract during a typical work week (40 h) during winter (blue) ands (red).

Results of UFP particle deposition modeling with the Multiple-Path Particle Dosimetry (MPPD) model are shown in Figure 5. Figure 5a shows the percentage of the UFP aerosol deposited in various lung regions. As shown, the total percentage of inhaled UFPs deposited in the overall region of the lungs was estimated to be 43.91% with 5.17% to be deposited in the head region, 15.13% in the tracheobronchial (TB) region, and 23.61% in the pulmonary region. These results indicate that almost half of the inhaled UFP aerosol would be deposited in various regions of the respiratory tract. Furthermore, it is of significant concern that the highest deposition percentage is in the pulmonary region (23%). These results further support earlier studies indicating the ability of UFP to penetrate deep in the lungs [82,83].

Figure 5b shows the mass of UFP deposited in various respiratory regions during a 40 h working week during the winter and summer. For winter UFP concentrations, the total UFP deposition in the respiratory system was 19.84 µg. Of this, 2.34 µg was estimated to be deposited in the head region. An amount of 6.84 µg was estimated to be deposited in the tracheobronchial region and 10.67 µg was estimated to be deposited in the pulmonary region. During the summer, the deposition levels increased: a total of 28.36 µg was expected to be deposited in the lungs, 3.34 µg was estimated to be deposited in the head region, 9.78 µg was estimated to be deposited in the tracheobronchial region, and 15.25 µg was estimated to be deposited in the pulmonary region.

### 3.7. Potential Health Implications for Garage Workers

The levels of UFP detected in this study are a cause for concern for the individuals exposed to the garage conditions. The implications of these (~10^4^ particles/cc) levels of UFPs are explored in a few studies, as detailed by a recent review of the implications of UFP in human health [84]. For example, Lane et al. and Noller et al. reported positive non-significant association between UFPs exposures of 10^4^ particles/cc levels from traffic-related activities and inflammatory biomarkers (hsCRP, IL-6, and TNFRII), ischemic heart disease (IHD), and cerebrovascular disease [85,86]. In a study by Hachem et al., Black Carbon (a major component of vehicular UFP) concentrations were assessed inside taxi vehicles for potential exposure to drivers and measured to be 2.2 to 3.9 μg/m^3^, which is a range of concentrations similar to the UFP concentrations of 2.51 μg/m^3^ and 3.59 μg/m^3^ observed in this study [87]. It was observed in the Hachem et al. study that such levels of BC increased the odds of acute respiratory effects (nasal irritation, impairment of lung function) in the drivers, thus linking such UFP exposure levels to negative health effects.

Apart from the size- and concentration-based implications, the chemical characterization of the UFPs collected has revealed the presence of high molecular weight PAHs, with the three largest PAH species, i.e., Benzo (ghi)perylene, Indeno (123cd)pyrene and Benzo (b+j)fluoranthene detected having carcinogenic effects. It should be noted that during the wintertime period (Figure 4c), PAH species, characterized as group 1, 2A, and 2B carcinogens by IARC, comprised 39% of the total PAH levels. This percentage of carcinogenic PAHs is higher compared to UFP studies in a road tunnel [88]. During summertime, this percentage is lower (7%); however, the concentration of the IARC PAHs is at the same levels (0.57 and 0.64 ng m^−3^, for winter and summer, respectively) [88]. Benzo (*ghi*)perylene has been shown to have toxic effects on the NL-20 human bronchial cell line [89,90]. Indeno (1,2,3-cd)pyrene has been shown to enhance allergic lung inflammation in a mouse model [91]. Retene is known to induce oxidative stress and cell death in cellular lung models [92]. To estimate the carcinogenic risk from the inhalation of particle-bound PAHs, the toxic equivalent factor (TEF) approach was used and a calculation of BaPeq was made according to the study of Taghvaee et al. [51]. PAH TEFs were selected based on the study of Li et al. [93]. Even though the PAH levels are higher during summer, the calculated BaPeq for UFP in this study presents no seasonal variability with values equal to 0.38 ng m^−3^ and 0.35 ng m^−3^ for the winter and summer period, respectively.

Furthermore, the significant amount of UFP deposited in the pulmonary region over a 40 h week as described above raises concerns about potential health implications for those working in garages, given the presence of toxic metals on the UFPs. Recently, the American Thoracic Society and the American Academy of Allergy, Asthma, and Immunology have emphasized the importance of research on potential adverse health effects of traffic-related UFP exposure [34,35]. Despite emerging evidence linking UFPs to respiratory and other diseases, their influence over endogenous resolution mechanisms and other effects remains completely unknown at this time. Therefore, there is a need to perform mechanistic studies to further understand the toxicological properties of the UFPs in garage environments and those from traffic-related exposure in general. The UFP collected from this study will be used in future toxicological studies by the authors, to understand the effect of these traffic-related UFPs on the resolution of inflammation, along with other endpoints.

## 4. Conclusions

In conclusion, the present study demonstrates the presence and the potential for deleterious health effects from exposure to UFP sampled during the winter and summer from a garage facility. The findings confirm the complex physicochemical properties of these UFPs. The major novelty of this study involves the identification and detailed characterization of PAHs obtained from the UFP, which have been shown to have carcinogenic and other negative health effects, using state-of-the-art methods. The computational modeling conducted with UFP levels measured during the winter and summer seasons indicated that microgram levels of UFPs would be deposited deep in the pulmonary alveolar region during a 40 h workweek, indicating a substantial health risk to any workers in the garage. To the best of the authors’ knowledge, this is a first of its kind study which assesses the risk of UFP exposure to the particular occupational group of garage workers and thus emphasizes the need to develop control measures for UFP exposure which are currently lacking.

## Data Availability

The original contributions presented in this study are included in this article; further inquiries can be directed to the corresponding author.
